# The Gold-headed Cane

**Published:** 1860-01

**Authors:** 


### Editors' Table.

The Gold-headed Cane.—We shall not be accused of imputing to them a lack of biographical lore, if we suppose that a majority of our readers have never heard of a small volume, entitled "The Gold-headed Cane," which professes to be the reminiscences of this at one time constant accessory and companion of every physician, when in full costume, and going his professional rounds.

"Physic of old, her entry made
Beneath the immense full bottom's shade,
While the gilt cane with solemn pride,
To each sagacious nose applied,
Seemed but a necessary prop,
To bear the weight of wig at top."

The quondam editor, in a most commendably short prefatory "Notice," gives a clue to the brief history, by telling us: "A short time before the opening of the New College of Physicians, [London,] Mrs. Baillie presented to the learned body a Gold-headed Cane, which had been successively carried by Drs. Radcliffe, Mead, Askew, Pitcairn, and her own lamented husband. The arms of these celebrated physicians are engraved on the head of the Cane, and they form the vignettes of the five chapters into which this volume is divided."

The Cane begins its autobiography by saying, that "It was deposited in a corner closet of the Library, on the 24th of June, 1825, the day before the opening of the New College of Physicians, with the observation, that I was no longer to be carried about, but to be kept among the relics of that learned body." After having been closely connected with medicine for a century and a half, it was natural that the gold-headed cane, like other celebrities, should employ its leisure in recording the most striking scenes it had witnessed. "Of my early state and separate condition," says the narrator, "I have no recollection whatever; and it may reasonably enough be supposed, that it was not till after the acquisition of my head that I became conscious of existence and capable of observation." The first consultation at which the Cane was present, was when its master, Dr. Radcliffe, was sent for, in the autumn of 1689, by the king, William the Third, then living at Kensington House. "We were ushered in, through a suite of several rooms, plainly but handsomely furnished, by Simon de Brienne; and it seemed to me that the Doctor assumed a more lofty air, and walked with a firmer step, and I was conscious of a gentle pressure of his hand as he stopped and gazed for a moment on the likeness of the founder of the College of Physicians, Dr. Linacre, painted by Holbein, which was hanging in one of the rooms, among royal portraits of the Henrys, and several other of the kings and queens of England and Scotland.

"On entering the sick chamber, which was a small cabinet in the south-east angle of the building, called the

Writing Closet, a person of a grave and solemn aspect, apparently about forty years of age, of a thin and weak body, brown hair, and of middle stature, was seen sitting in an arm-chair, and breathing with great difficulty. The naturally serious character of the king (for it was his majesty, William the Third,) was rendered more melancholy by the distressing symptoms of asthma, the consequence of the dregs of the small-pox, that had fallen on his lungs." Dr. Radcliffe was not inclined to play the courtier on the occasion, and he addressed his royal patient in the following terms: "May it please your majesty, I must be plain with you, sir; your case is one of danger, no doubt, but if you will adhere to my prescriptions, I will engage to do you good. The rheum is dripping on your lungs, and will be of fatal consequence to you, unless it be otherwise diverted." The insinuation in the last remark was not complimentary to the king's body physicians, Bidloo and Laurence, nor was it in good taste nor conformable with good ethics. The indication to be fulfilled was to procure a flow of saliva, but by what means we are not informed. This, however, having been obtained, the king was entirely relieved, and "a few months afterwards he fought the battle of the Boyne."

Before leaving the palace, Dr. Radcliffe waited upon her majesty the queen, whose affability and cultivated mind are deservedly praised on the occasion. This estimable lady died five years afterwards of the small-pox —an incident which, by reminding us of the protection from this fatal and loathsome disease, afforded by vaccination, to both king and peasant, ought to make us proud of a profession which numbers a Jenner in its ranks. The death of his wife was keenly felt by the generally impassive king. Radcliffe, in a subsequent year, 1697, after the treaty of Ryswick, on being summoned to the royal presence, expressed himself with startling bluntness, by telling his patient that he must not be buoyed up with hopes that his malady would soon be driven away. "Your juices are all vitiated, your whole mass of blood corrupted, and the nutriment for the most part turned to water; but," added the Doctor, "if your majesty will forbear making long visits to the Earl of Bradford, (where, to tell the truth, the king was wont to drink very hard,) I'll engage to make you live three or four years longer; but beyond that time no physic can protract your majesty's existence." After an interval of three years, the king, while hunting, fell from his horse and broke his right clavicle, near the acromion. "This occurred in the neighborhood of Hampton Court; but the French surgeon Ronjat was at hand, and soon reduced the fracture. But when he wanted to bleed his majesty, a new obstacle arose, for it was necessary not only to have the sanction of some one of the court physicians, but also the authority of the privy council for the performance of this operation." Disregarding the recommendation of his professional advisers, that he should keep perfectly quiet, the king insisted on returning to Kensington; but on arriving there a new difficulty occurred, showing that royalty enjoys no special privileges over the commonalty, in exemption from the annoyances growing out of differences between doctors, on the score of diagnosis and of medical etiquette. A discussion arose between Bidloo and the surgeon, as to whether there had really been any fracture or not. "Ronjat strongly maintained the affirmative, the Dutch doctor as stoutly denied it. This point was at length settled, when a new difference of opinion occurred as to the mode of applying the bandages. Bidloo wished himself to apply them, but the surgeon said no. 'You are here either in the character of a physician or in that of a surgeon; if the former, you have nothing to do with bandages; if the latter, *c'est moi qui suis le premier chirurgeon du Roi.*' After the death of the king, a paper war took place, and the various arguments and statements advanced by each party were frequently mentioned in societies where I was present, for luckily my master had no share in these disputes." We pass over the remains of this digression, in which the character of King William is sketched, and an incident related of his eating up all the green peas, "then newly come in, without even once offering this rarity to his royal consort, or guest," her sister, the Princess Anne, afterwards queen.

To return to the eminent bearer of the gold-headed cane, Dr. Radcliffe, who was sorely tried in his first courtship and betrothal. The lady was an only child, rich, "and with a tolerable share of personal charms." "Matters seemed to proceed prosperously, and everything promised a consummation of my master's happiness; when, one evening, he returned late to his home, obviously much discomposed." One may well imagine his mortification, when we learn its cause, which was no less than a discovery that his betrothed had given her affections and her person to another man, of which fact she began to show proof beyond dispute. Well might the Doctor exclaim to himself: "Mrs. Mary is a very deserving gentlewoman, no doubt; but her father must pardon me if I think her by no means fit to be my wife, since she is or ought to be another man's already!" An occurrence of this nature was not adapted to the removal of Radcliffe's aversion to matrimony, although, later in life, at the maturer age of sixty, he did a wooing go. He is described as having an elevated forehead, hazel eyes, cheeks telling of the good cheer of former days; if anything, a little too ruddy; a double-chin, a well-formed nose, and a mouth round which generally played an agreeable smile. Fortunately for literature and science, toward the advancement of which he left such noble legacies, the lady did not smile on him. "Suffice it to say," says our gold-headed biographer, "he was lampooned, proved unfortunate in his suit, and was styled by the wicked wits of the day, 'the mourning Esculapius,' 'the languishing, hopeless lover of the divine Hebe, the emblem of youth and beauty.'" Prince Eugene, the friend of Marlborough, when on a visit to London, dined with Radcliffe, who determined that there should be "no ragouts, no kickshaws of France," but treating his guest as a soldier, he had his "table covered with barons of beef, jiggets of mutton, and legs of pork." A portrait of the Prince is drawn on the occasion. Reference is made here, for the first time, to Dr. Mead, who is introduced in the following dialogue. "He lived then in Austin Friars, and we found him, one morning, in his library reading Hippocrates.

"*Radcliffe* (taking up the volume of the venerable father of physic.) 'What! my young friend, do you read Hippocrates in the original? Well, take my word for it, when I am dead you will occupy the throne of physic in this great town.'

"*Mead.* 'No, sir; when you are gone, your empire, like Alexander's, will be divided among many successors.'" Mead had already written his treatise on Poison. Many rooms of his small house were filled with books, and the two doctors indulged in a long chat. Mead was very lively and entertaining, related several anecdotes of things which he had seen abroad; and described with great animation his joy in finding the *Tabula Isiaca*, in a lumber room, in Florence. "Upon this subject, my master asked many questions, and appeared much struck with the advantage of foreign travel to a physician."

Radcliffe encountered much ill-deserved odium for not joining Mead and the household physicians in visiting Queen Anne, in the last days of her fatal illness; and the death of the sovereign was laid to him. He was, however, unable to attend, being himself confined to his country-house, at Cashalton, in Surrey, by the gout, which had seized on his head and stomach. Soon after this time, as we read in the reminiscences of the Gold-headed Cane, he invited Mead to dine with him, and, after declaring his intention to withdraw himself from practice, he handed over to the latter the mute but valued companion in his daily rounds, and in a measure the medical sceptre itself. He gave, at the same time, a short sketch of his professional life. "My books," he says, "have always been few, though well chosen. When I was at the University, a few vials, a skeleton, and an herbal, chiefly formed my library." "I have always endeavored to discountenance the attempts of quacks and intermeddlers in physic, and by the help of Providence I have succeeded most wonderfully." He refers to his services at court, and the requital by the King, (William,) who, on one occasion, ordered him five hundred guineas (upwards of $2500) out of the privy purse, for the cures of Bentinck and Zulestein. By attendance on his majesty, Radcliffe "gained, one year with another, more than six hundred pounds ($3000) per annum." Dr. Gibbons was a lucky man in being the neighbor of Dr. Radcliffe, whose overflow of business, to which he was unable to attend, brought the former a thousand pounds a year. Speaking of his failing health, and the short time he has to live, the retiring chief says: "The time is, I am afraid, barely sufficient to repent me of the idle hours which I have spent in riotous living; for I now feel, in the pain which afflicts my nerves, that I am a martyr to excess, and am afraid that I have been an abettor and encourager of intemperance in others."

Dr. Radcliffe died November 1st, 1714. His eulogy may be written in a few lines. The figures in it are not those of rhetoric, nor of poetry; but they carry with them a fullness of thought, a farsightedness, a force, and an animation, more convincing and more enduring in their effects than the finest prize poem or oration. They have elevated science, expanded literature, imparted new hopes to the desponding, raised the sick and the infirm from the bed of suffering, and snatched many from the grasp of death itself. Radcliffe wrought all these wonders by his last will and testament. He left his Yorkshire estate to the Master and Fellows of University College, Oxford, forever, in trust for the foundation of two traveling fellowships, the overplus to be paid to them for the purposes of advowsons for the members of said college.

Five thousand pounds for the enlargement of the building of University

College, where he himself had been educated.

Forty thousand pounds ($200,000) for the building of a Library at Oxford.

Five hundred pounds yearly, forever, toward mending the diet of St. Bartholomew's Hospital.

After the payment of these bequests, and some legacies to various individuals mentioned in the will, he gave to his executors in trust, all his estates, in four counties, to be applied to such charitable purposes as they in their discretion should think best; but no part thereof to their own use or benefit. The result of this last bequest is seen in the Observatory and Public Infirmary at Oxford, both of which were erected out of the funds of Dr. Radcliffe's estate by the trustees of his will. The Radcliffe Library, which is, perhaps, the most beautiful building in Oxford, was finished in 1749. It has been appropriated by a resolution of the trustees to the reception of books on medicine and natural history. The streams of benevolence still flow in other and smaller channels from the sources kept open by the faithful and enlightened guardians of the Radcliffe estate-funds, in the exercise of the discretionary power with which they are intrusted.

